# Involvement of a Novel Class C Beta-Lactamase in the Transglutaminase Mediated Cross-Linking Cascade of *Streptomyces mobaraensis* DSM 40847

**DOI:** 10.1371/journal.pone.0149145

**Published:** 2016-02-17

**Authors:** Stephan Zindel, Vera Ehret, Marina Ehret, Madeleine Hentschel, Samantha Witt, Andreas Krämer, David Fiebig, Norbert Jüttner, Sabrina Fröls, Felicitas Pfeifer, Hans-Lothar Fuchsbauer

**Affiliations:** 1 Department of Chemical Engineering and Biotechnology, University of Applied Sciences of Darmstadt, Darmstadt, Germany; 2 Department of Biology, Technische Universität Darmstadt, Darmstadt, Germany; CNR, ITALY

## Abstract

*Streptomyces mobaraensis* DSM 40847 secretes transglutaminase that cross-links proteins via γ-glutamyl-ε-lysine isopeptide bonds. Characterized substrates are inhibitory proteins acting against various serine, cysteine and metalloproteases. In the present study, the bacterial secretome was examined to uncover additional transglutaminase substrates. Fractional ethanol precipitation of the exported proteins at various times of culture growth, electrophoresis of the precipitated proteins, and sequencing of a 39 kDa protein by mass spectrometry revealed the novel beta-lactamase Sml-1. As indicated by biotinylated probes, Sml-1, produced in *E*. *coli*, exhibits glutamine and lysine residues accessible for transglutaminase. The chromogenic cephalosporin analogue, nitrocefin, was hydrolyzed by Sml-1 with low velocity. The obtained K_m_ and k_cat_ values of the recombinant enzyme were 94.3±1.8 μM and 0.39±0.03 s^-1^, respectively. Penicillin G and ampicillin proved to be weak inhibitors of nitrocefin hydrolysis (K_i_ of 0.1 mM and 0.18 mM). Negligible influence of metals on β-lactamase activity ruled out that Sml-1 is a Zn^2+^-dependent class B beta-lactamase. Rather, sequence motifs such as SITK, YSN, and HDG forming the active core in a hypothetical structure may be typical for class C beta-lactamases. Based on the results, we assume that the novel transglutaminase substrate ensures undisturbed growth of aerial hyphae in *Streptomyces mobaraensis* by trapping and inactivating hostile beta-lactam antibiotics.

## Introduction

Distinct streptomycetes, among them *Streptomyces mobaraensis* DSM 40847, produce extracellular transglutaminase (TGase, P81453) that most likely evolved independently from its mammalian and phytogenic counterparts [1]. Besides missing sequence homology, activity in the absence of calcium ions is one of the most prominent features of the microbial enzyme. TGases contain cysteine, histidine and aspartate in close vicinity to each other, thus forming the proton relay of a catalytic triad [2,3]. These amino acids are likewise situated in a deep cleft of the flat protein disk of the microbial enzyme [4]. Arrangement in the crystal, however, revealed closer proximity of cysteine to aspartate than to histidine, suggesting modified proton transfer and catalysis. Moreover, substrate proteins have open access to microbial TGase because β-sandwich and β-barrel domains which flank the catalytic domain of the mammalian enzymes are absent. The microbial enzyme only contains a 45mer propeptide that is removed by the transglutaminase activating metalloprotease (TAMP, P83543) and an Ala-Pro-specific tripeptidylaminopeptidase (P83615) during culture of *S*. *mobaraensis* [5–7].

The most common reaction catalyzed by TGases is cross-linking of proteins *via* N^ε^-(γ-glutamyl)lysine isopeptide bonds [2]. Accessibility of the enzyme to water-exposed substrate glutamines is thought to be decisive in forming the acyl enzyme complex and transferring the γ-glutamyl moiety onto protein-bound lysines. In addition, incorporation of primary amines into glutamine donor proteins and *endo*-glutamine deamidation may be the result of TGase activity [8]. Hydrolysis of substrate glutamines by TGase or intramolecular cross-linking seem to be the most preferred reactions in submerged culture of *S*. *mobaraensis*, most likely due to shearing forces that prevent the formation of the ternary protein complex [8–10].

The physiological substrates of microbial TGase are inhibitory proteins that are secreted into the culture medium of *S*. *mobaraensis* [8–10]. The *Streptomyces* subtilisin and TAMP inhibitor (SSTI, P83544) belongs to the well-characterized family of *Streptomyces* subtilisin inhibitors (SSI) or SSI-like proteins, respectively [6,8]. SSTI is a homodimer consisting of 2x14 kDa subunits and has binding sites for serine proteases such as subtilisin (P00782) and trypsin (P00760), and metalloproteases such as TAMP. It may be plausible that TGase diminishes the amount of SSTI by cross-linking and the formation of highly polymerized aggregates in the bacterial cell wall. As result, enhanced activity of TAMP may contribute to more activated TGase in a positive feedback circuit. There are two additional substrates of TGase characterized, the *Streptomyces* papain inhibitor (SPI, P86242) and the dispase autolysis inducing protein (DAIP, P84908) [9,10]. In contrast to the SSI-like SSTI, genes encoding SPI are absent in most of the streptomycetes whose genome sequences have been determined. The 12 kDa SPI inhibits cysteine and serine proteases such as papain (P00784), gingipains (P28784, Q51817) or trypsin in nanomolar concentrations, and growth of various bacteria [10,11]. DAIP is unique and not related to any other characterized protein. The 37 kDa protein destroys neutral metalloproteases such as bacillolysin (P29148) and thermolysin (P00800) by triggering autolysis.

In the present report, we describe a secretome analysis procedure that allows the detection of additional TGase substrates in submerged cultures of *S*. *mobaraensis*. Sequencing of a 39 kDa protein by mass spectrometry, production in *E*. *coli* and further characterization of the recombinant protein revealed a novel class C β-lactamase exhibiting glutamine and lysine cross-linking sites for the intrinsic transglutaminase.

## Materials and Methods

### Culture procedures

*Streptomyces mobaraensis* DSM 40847 (formerly *Streptoverticillium mobaraense*) was obtained from the German Collection of Microorganisms and Cell Cultures (DSMZ) in Braunschweig (Germany). For maintenance and inoculation of submerged cultures, spores were allowed to grow on glucose-yeast-malt (GYM) agar at 28°C as described [12]. Re-inoculation occurred every month. Colonies grown on GYM for 30-40 d were used to inoculate starch mineral salt media for the production of enzymes and enzyme inhibitors [12]. *Escherichia coli* BL21(DE3) RIL (F¯ *ompT hsdS*(r_B_¯ m_B_¯) *dcm*^+^ Tet^r^ *gal* λ(DE3) *endA* Hte [*argU ileY leuW* Cam^r^]), used as expression host, was obtained from Merck-Millipore, Darmstadt (Germany). Transformants were grown on Luria-Bertani (LB) medium. Ampicillin (100 μg/ml) was added as required (LB_amp_).

### Screening procedure, production of the beta-lactamase by *S*. *mobaraensis*, purification and sequencing

Culture of *S*. *mobaraensis* was performed in starch mineral salt medium at pH 7 and 28°C up to 96 h [12]. Samples were taken every 12 h, starting at 24 h, and centrifuged. Supernatants on ice were gradually combined with -18°C cold ethanol up to 80 vol% (10 vol% increments) and separated by 12.5% SDS PAGE. The 50-60 vol% ethanol precipitate of a 68 h culture (110 ml) was separated by Fractogel EMD SO_3_¯ chromatography at pH 4 using 0–1 M NaCl in 50 mM acetate (S1 Fig) [9]. The 39 kDa protein was cut out from a Coomassie-stained 12.5% SDS polyacrylamide gel and sequenced by nanoLC-ESI-MS/MS. The protein databases were searched using 22 sequenced peptides and MASCOT resulting in a score of 397 (PANATecs, Heilbronn, Germany).

### Codon optimization, plasmids, cloning, transformation, and purification of the recombinant beta-lactamase

The gene encoding the β-lactamase Sml-1 was optimized by GenScript (Hongkong, China) to enhance efficiency of gene translation in *E*. *coli* (S2 Fig). The 3’-end of the synthesized gene (*bla*_Sml-1,opt_) was extended by additional codons for methionine (ATG) and glycine (GGG). The 1085 bp coding sequence of *bla*_Sml-1,opt_ was cloned into pET-22b(+) *via* restriction sites *NcoI* and *XhoI*, and *E*. *coli* BL21(DE3) RIL were transformed with the resulting construct using the heat-shock procedure. Recombinant clones were selected by colony PCR, re-produced on LB_amp_ and stored as glycerol stocks at -80°C. Transformants were capable to produce recombinant Sml-1 (*r*Sml-1) extended at N- and C-termini by pelB leader signal peptide for secretion into the periplasm and an additional Gly-Leu-Glu-(His)_6_ peptide, respectively. Production of *r*Sml-1 was performed by incubation of verified clones in 1 l LB_amp_ at r.t. and 37°C over-night and induced with 1 mM IPTG. Cells were harvested the following day, washed with 50 mM NaCl in 50 mM Tris-HCl pH 8, and raptured by sonication. The cellular debris were removed by centrifugation (20,000 ***g***, 25 min, 4°C), and the supernatant was mixed consecutively with 40 vol% and 70 vol% ethanol while cooling. Final purification was performed by immobilized metal ion affinity chromatography of the 70 vol% ethanol precipitate using Chelating Sepharose Fast Flow (bed volume of 7 ml), 50 mM NaCl and 30 mM imidazole in 20 mM Tris-HCl pH 8 and a linear gradient of 30–500 mM imidazole in the same buffer (S3 Fig).

### Beta-lactamase assay according to O'Callaghan *et al*. [13]

Activity of wildtype Sml-1 and recombinant *r*Sml-1 was determined by adding 10-100 μM enzyme (20 μl) to 1 mM nitrocefin (20 μl), 0.5 M NaCl in 0.5 M Tris-HCl pH 7 (20 μl) and 140 μl water (final volume of 200 μl). Increase in absorbance was monitored continuously up to 60 min at 492 nm. The extinction coefficient for 200 μl, obtained by complete nitrocefin hydrolysis after 30 min, was 9.430 ml μmol^-1^ (S4 Fig). Enzyme activity was defined as the formation of one micromole hydrolyzed nitrocefin per min and ml. Other buffers such as citrate (pH 3–6.5), phosphate (pH 6–8), Tris-HCl (pH 7–9), and glycine (pH 9–10) were used in equal concentration and composition to study pH optimum (S5 Fig). Metal ions, serine protease inhibitors, penicillin G, ampicillin, and Ac_2_Lys-DAla-DAla were added to the master mix as required. Kinetic and IC_50_ data were obtained by varying the amount of nitrocefin, penicillin G and ampicillin and fitted by Graphpad Prism 5. The IC_50_ data were used to calculate K_i_ values according to Cheng and Prusoff [14]. For display of β-lactamase in sporulating *S*. *mobaraensis*, colonies on GYM agar were covered with 6 ml 0.15 mM nitrocefin and shaken for 15 min and 30 min. Absorbance of supernatants (200 μl) was monitored at 492 nm as described above.

### Determination of DD-carboxypeptidase and DD-transpeptida-se activity

The tripeptide Ac_2_Lys-DAla-DAla (30 mM) and 50 mM NaCl in 50 mM phosphate pH 7, even in combination with 30 mM glycine ethyl ester, were incubated with 50 μM *r*Sml-1 at 37°C for up to 24 h. Reaction mixtures were spotted onto silica TLC plates and developed using ethyl acetate/pyridine/acetic acid/water 30:20:6:11. Compounds were visualized by consecutive staining using ninhydrin (indicating released D-alanine and glycine ethyl ester) and vanillin/sulfuric acid (indicating Ac_2_Lys-DAla-DAla, Ac_2_Lys-DAla, Ac_2_Lys-DAla-GlyOEt).

### Other enzyme and inhibitor assays

Activity of microbial transglutaminase was determined by incorporation of hydroxylamine into N^α^-carbobenzoxy-L-glutaminylglycine and addition of ferric chloride in acid solution [12]. For measuring proteolytic activity, degradation of casein was observed by monitoring the absorbance of supernatants at 280 nm upon addition of 10% trichloroacetic acid as described [8]. Inhibitory proteins such as SSTI and SPI were indicated by preceding incubation with subtilisin and papain [8,10].

### Other experimental procedures

Transglutaminase mediated biotinylation of *r*Sml-1 using monobiotinylcadaverine (MBC, probe for glutamine residues), 1-N-biotinyl-6-N’-(carbobenzoxy-L-glutaminyl glycyl)hexanediamine (ZQGB), and N^α^-biotinyl-Thr-Val-Gln-Gln-Glu-Leu-OH (BTVQQEL), both probes for lysine residues from Zedira (Darmstadt, Germany), was performed as described (S6 Fig) [8]. Protocols for SDS polyacrylamide gel electrophoresis, silver- or Coomassie-staining, and Western blotting are described in ref. [5]. For fluorescence staining, rabbit antibodies against pro-transglutaminase, DAIP, SSTI, and IRDye800CW labelled goat (anti-rabbit-IgG) antibodies and IRDye800CW labelled streptavidin (Li-Cor, Bad Homburg, Germany) were used according to the manufacturer’s protocol. Gels and blots were photographed using BioDocAnalyze (Biometra, Göttingen, Germany) and Odyssey (Li-Cor).

### Databases

Sequence and experimental data of the β-lactamase from *S*. *mobaraensis* are available in the UniProt database under the SPIN ID number SPIN200003201, model data of the β-lactamase from *S*. *mobaraensis* in the PMDB database under the accession number PM0079826.

## Results

### Extracellular proteins from *Streptomyces mobaraensis*

**Fig 1:**
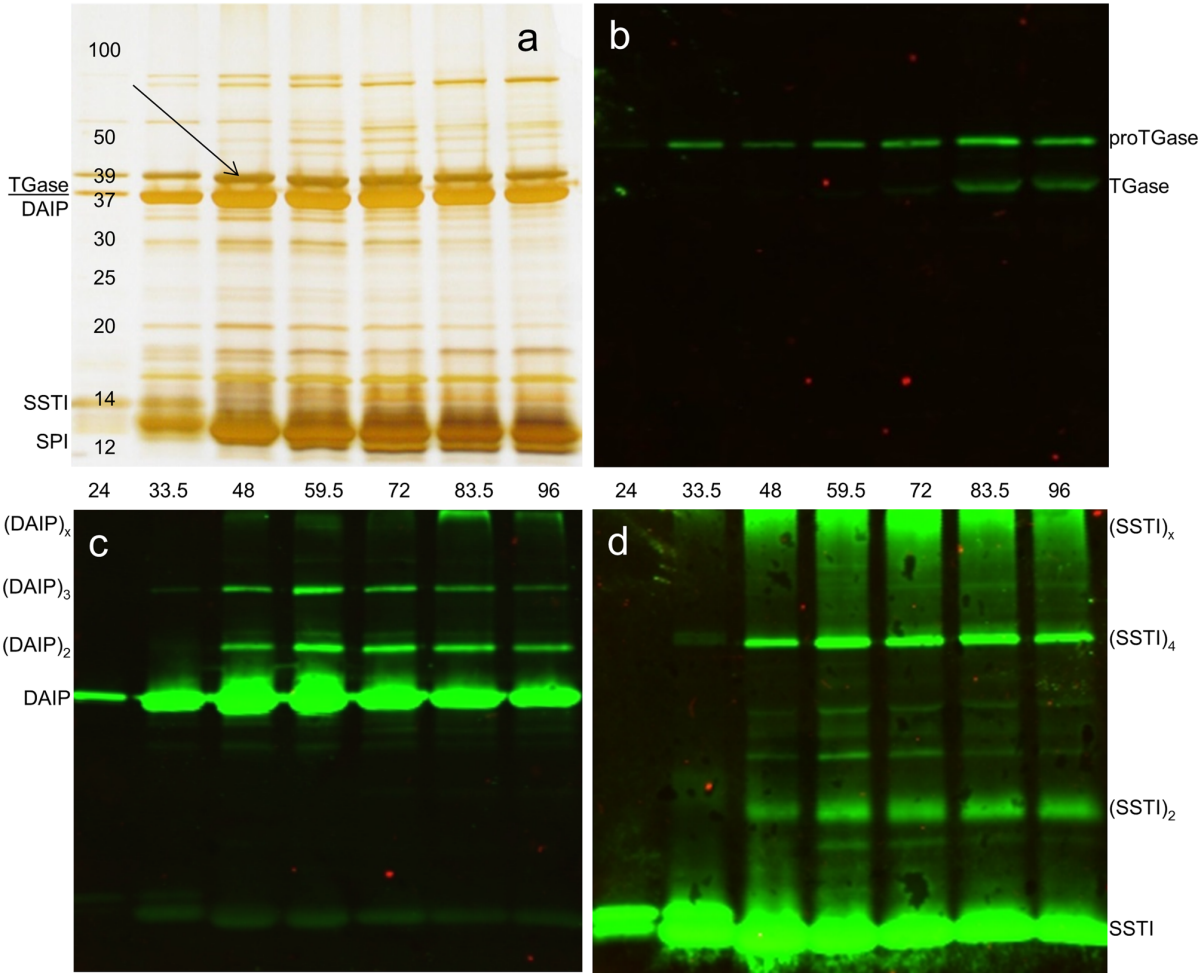
Discovery of the novel 39 kDa protein (β-lactamase Sml-1). *S*. *mobaraensis* was allowed to grow submerged in a starch mineral salt medium at 28°C for the indicated hours. Upon removal of cell aggregates, 50–60 vol% ethanol precipitates were separated by 12.5% SDS polyacrylamide gel electrophoresis: a, silver-stained gel; b-d, Western blots visualized by antibodies against proTGase (b), DAIP (c), and SSTI (d). The studied protein at 39 kDa is indicated by an arrow.

Transglutaminase and the formerly characterized TGase substrates are soluble proteins which are secreted by *S*. *mobaraensis* because of impaired aerial mycelium formation under submerged culture conditions. Thus, we have studied extracellular proteins (secretome) at various times of culture growth to determine additional substrates of the cross-linking enzyme. Samples were drawn every twelve hours and fractioned by ethanol up to final concentrations of 80 vol%. The precipitates were analyzed by electrophoresis and Western-blotting (Fig 1).

Strong bands at 12–14 kDa (SSTI, SPI) and at 37 kDa (DAIP) indicated the early export of TGase substrates by *S*. *mobaraensis* between 24 hours and 48 hours. In contrast, highest amounts of TGase, displayed by zymogen and mature enzyme, were obtained after 60–72 hours of culture (not shown). While the papain inhibitor (SPI) was detected in nearly all precipitates, the majority of TGase, DAIP (dispase autolysis inducing protein) and SSTI (*Streptomyces* subtilisin and TAMP inhibitor) lost solubility after addition of 30–50 vol% (TGase) and 50–70 vol% ethanol (DAIP, SSTI), respectively. The 50-60 vol% ethanol precipitate contained only traces of the cross-linking enzyme (Fig 1a and 1b). However, besides DAIP and SSTI monomers, TGase mediated aggregates, even formed under mechanical stress, were detected by immunostaining (Fig 1c and 1d). Within the first 36 hours of culture, when TGase is not yet activated, oligomers of the substrate proteins were scarcely visible. Only SSTI showed two adjacent bands, most likely the result of N-terminal truncation by the Ala-Pro-specific tripeptidylaminopeptidase [7]. Appearance of aggregates then indicated the time of TGase activation. Unknown proteins emerged between 16 kDa and 20 kDa, and at 39 kDa. The 39 kDa protein (p39) was purified for additional investigations (Fig 1a, arrow).

### Identification of the extracellular 39 kDa protein from *S*. *mobaraensis*

Purification was performed using cell-free culture supernatants from S. *mobaraensis*. Fractogel EMD SO_3_¯ chromatography of the 50–60 vol% ethanol pellet at pH 4 resulted in five well separated protein peaks (S1 Fig). The 39 kDa protein eluted at 0.5–0.6 M NaCl and was only contaminated by considerable amounts of small proteins forming at least two bands at 10–12 kDa (S1 Fig, insert). Inhibition of papain and subtilisin suggested SPI and SSTI were the impurities of the p39 fractions. Two additional separation procedures using Superdex 75 and Fractogel EMD TMAE were performed to remove SPI and SSTI. However, tiny amounts of a protease, most likely inhibited by SPI or SSTI before, fragmented the 39 kDa protein during size exclusion chromatography (not shown) or heating in SDS upon TMAE chromatography (S7 Fig). Hence, the unknown protein was separated from SPI and SSTI by electrophoresis, cut out and sequenced using nanoLC-ESI-MS/MS.

**Fig 2:**
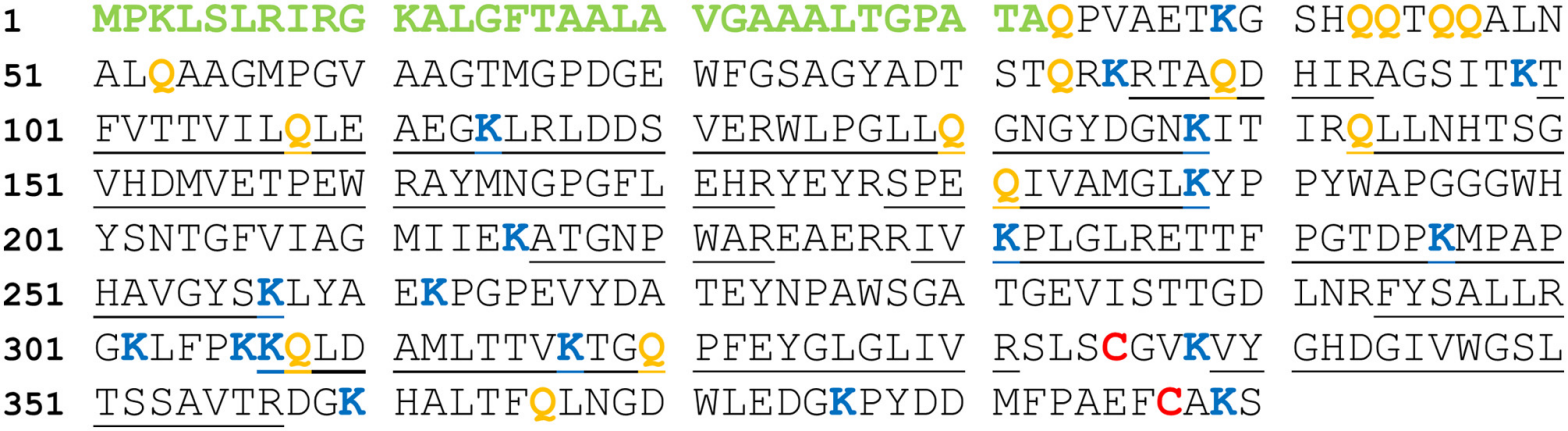
Sequence of the novel 39 kDa protein (β-lactamase Sml-1) from *S*. *mobaraensis*. Amino acids identified by nanoLC-ESI-MS/MS are underlined. Glutamines (15), lysines (19) and cysteines (2) of the mature enzyme, and the putative signal peptide are shown in orange, blue, red and green, respectively.

The analyzed 22 peptides, covering about 45% of a putative protein sequence from *S*. *mobaraensis* (M3B5M4), showed similarities to the low molecular mass penicillin binding proteins (LMM-PBP) (Fig 2). SignalP-4.1 prediction suggested truncation of the signal peptide by cleavage of the Ala32-Gln33 peptide bond and, thus, a calculated molecular mass of 39.2 kDa for mature p39. The sequence displayed two cysteines in addition, most likely forming a single disulfide bridge, and a high number of glutamine and lysine residues, important for cross-linking by transglutaminase.

### Recombinant production of the 39 kDa protein from *S*. *mobaraensis*

**Fig 3:**
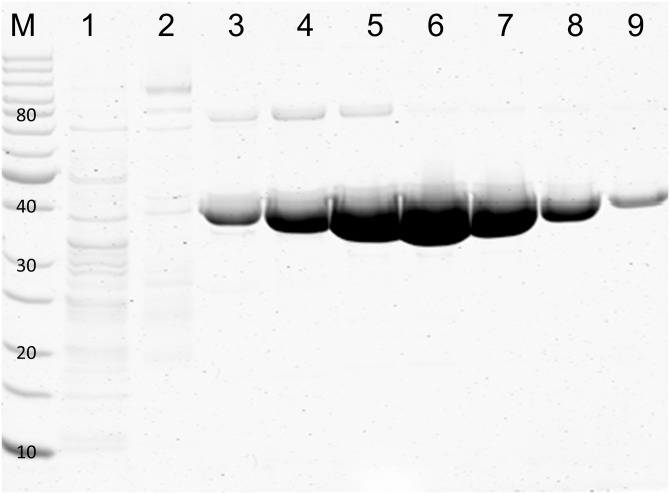
Protein pattern of the purified 39 kDa protein (β-lactamase Sml-1) from *S*. *mobaraensis* produced in *E*. *coli* BL21(DE3) RIL. Production was performed at ambient temperature. Upon cell lysis and centrifugation, proteins of the supernatant were precipitated by 40-70 vol% ethanol and separated by IMAC at pH 8 using a linear gradient of 30–500 mM imidazole: lane M, molecular weight marker mixture; lane 1, run-through; lanes 2–9, fractions after 94 ml, 115 ml, 117 ml, 119 ml, 121 ml, 123 ml, 125 ml, and 127 ml (S3 Fig).

A synthetic gene encoding the 39 kDa protein from *S*. *mobaraensis* was used to transform *E*. *coli* BL21(DE3) RIL *via* pET-22b(+) incorporation (S2 Fig). Extensions for a preceding signal peptide and a C-terminal hexahistidine tag allowed secretion into the periplasm and IMAC purification, respectively. Cultivation was performed in LB-medium at ambient temperature before IPTG was added. After cell separation and ultrasonic disintegration, supernatants were treated with 40 vol% ethanol resulting in nearly complete precipitation of *E*. *coli* proteins. Recombinant p39 (*r*p39) precipitated at 70 vol% ethanol and eluted from a 7 ml IMAC column at 230-270 mM imidazole (S3 Fig). The procedure yielded approximately 10 mg of highly purified *r*p39 per liter of culture, and IMAC fractions contained up to 12.8 mg/ml protein (Fig 3).

### Labelling of the recombinant 39 kDa protein by transglutaminase

**Fig 4:**
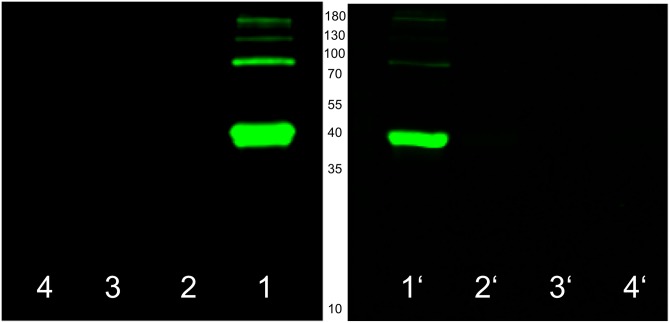
Biotinylation of the recombinant 39 kDa protein (β-lactamase Sml-1) *from S*. *mobaraensis* by microbial transglutaminase. Labelling of 10 μM *r*p39 was performed by 0.1 μM TGase mediated incorporation of the biotinylated hexapeptide BTVQQEL (left) or monobiotinylcadaverine (right), both 0.4 mM, in 0.1 M HEPES pH 7.5 overnight. Biotinylation was visualized using fluorescent streptavidin conjugates upon electrophoresis and protein blotting. Lanes 1/1’, labelling mixtures; lanes 2/2’, 3/3’, and 4/4’, controls without TGase, *r*p39 or the biotinylated probes, respectively.

Purified *r*p39 was used to determine cross-linking sites of microbial TGase by enzymatic incorporation of biotinylated probes such as monobiotinylcadaverine (MBC), 1-*N*-biotinyl-6-*N’*-(carbobenzoxy-L-glutaminylglycyl)diamidohexane (ZQGB) and N^α^-biotinyl-Thr-Val-Gln-Gln-Glu-Leu (BTVQQEL). The lysine equivalent MBC thereby allows the detection of glutamine residues but the glutamine peptides ZQGB and BTVQQEL indicate the presence of accessible lysines. After incubation for two hours, reaction mixtures were separated by SDS-PAGE, blotted onto nitrocellulose membranes and stained by streptavidin alkaline phosphatase conjugates that cause the formation of purple dyes from 5-bromo-4-chloro-3’-indolyl phosphate and nitro blue tetrazolium. Single protein bands at molar TGase *r*p39 ratios of 1:10 suggested biotinylation of substrate glutamines and lysines (S6 Fig). However, the degree of glutamine labelling was obviously low, and controls without *r*p39 showed autobiotinylation of the enzyme. In addition, high molecular weight aggregates of *r*p39 were absent. We assumed that the majority of accessible *r*p39 glutamines were hydrolyzed by TGase prior to incorporation of MBC and protein cross-linking. The labelling procedure was repeated at the reduced enzyme substrate ratio of 1:100 and prolonged incubation. Usage of a fluorescent streptavidin conjugate should only allow the attachment to protein-bound biotins without enzymatic signal amplification. Because of similar molecular masses and autobiotinylation, *r*p39 was separated from TGase by ethanol precipitation to avoid false-positive results. The additional experiment clearly confirmed that *r*p39 is a substrate of microbial TGase (Fig 4). Only in the presence of the enzyme, linkage of the biotinylated probes to *r*p39 occurred, and lysine labelling was stronger again. Moreover, the biotinyled hexapeptide BTVQQEL could not competitively prevent cross-linking of *r*p39 as suggested by the occurrence of high molecular weight aggregates.

### Specificity and activity of the 39 kDa protein

As outlined above, the novel transglutaminase substrate p39 is related to members of the large family of penicillin-binding proteins comprising β-lactamases, DD-carboxypeptidases, DD-transpeptidases, and peptidoglycan peptidases. Incubation of the recombinant protein with casein ruled out proteolytic activity, even excluding that the described fragmentation of p39 upon removal of SPI and SSTI was the result of autolysis. Furthermore, the amino-protected tripeptide Ac_2_Lys-DAla-DAla was used as equivalent of the glycan-bound pentapeptide Ala-*iso*Glu-*m*DAP-DAla-DAla to determine DD-carboxypeptidase and DD-transpeptidase activity [15]. In *Streptomyces*, the meso-diaminopimelic acid is linked to a single glycine that is transferred by DD-transpeptidases onto D-alanine after release of the terminal D-alanine, thus forming cross-bridges in the mature peptidoglycan [16]. We incubated the tripeptide with *r*p39 at pH 7 and separated the mixture by thin layer chromatography. In a similar attempt, glycine ethyl ester was added to determine DD-transpeptidase activity. In both cases, released D-alanine was not observed, not even after 24 hours, suggesting p39 is not a DD-carboxypeptidase or a DD-transpeptidase (results not shown). However, nitrocefin, a cephalosporin analogue for measuring β-lactamase activity [13,17], was hydrolyzed by *r*p39 indicated by increase in absorbance at 492 nm. Accordingly, we denote the novel TGase substrate as *Streptomyces mobaraensis* β-lactamase-1 (Sml-1, *r*Sml-1) afterwards.

### Secretion of the beta-lactamase Sml-1 by *Streptomyces mobaraensis*

A continuously running nitrocefin assay was established to verify secretion of Sml-1 by *S*. *mobaraensis*. Constant hydrolysis rates, indicated by linear increase in absorbance at 492 nm, were observed within the first 5–10 minutes at pH 7 after 3.5 μM *r*Sml-1 was added to 60-200 μM nitrocefin (S4 Fig). After 20 minutes, complete cleavage of the substrate was achieved. The endpoints were used to determine the molar extinction coefficient of 9.430 ml μmol^-1^ for 200 μl.

**Fig 5:**
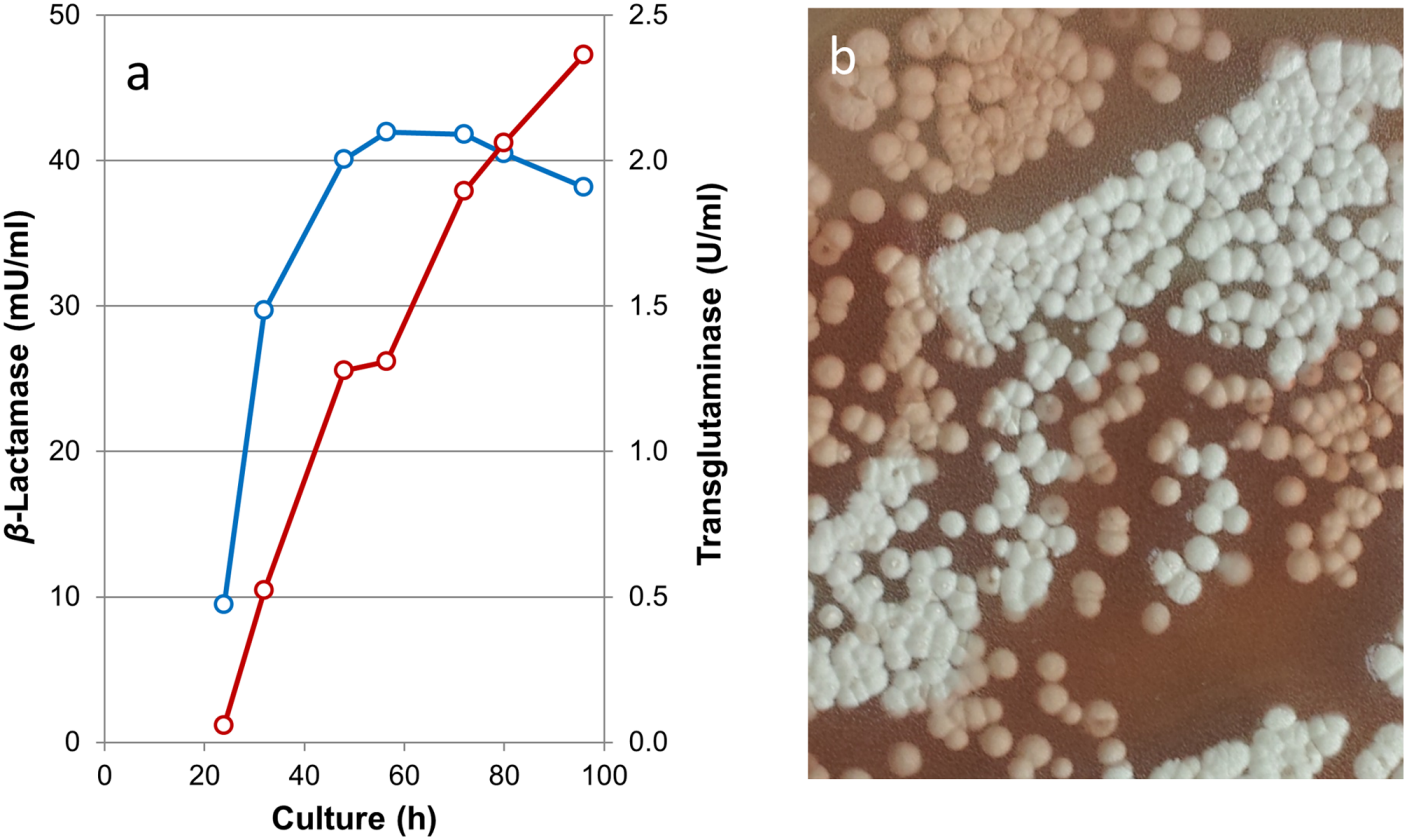
Secretion of the β-lactamase Sml-1 and transglutaminase from *S*. *mobaraensis*. (a) Cultivation was performed in a starch-mineral salt medium at 28°C. Activity of Sml-1 (blue) and TGase (red) was measured using nitrocefin and carbobenzoxy-L-glutaminylglycine/hydroxylamine, respectively, as described in the Materials and methods. (b) Sporulating surface colonies on GYM agar after 3d-culture growth were covered with nitrocefin and incubated for 30 min.

Next, export of Sml-1 and transglutaminase was studied by measuring the β-lactamase activity of Sml-1 in culture supernatants of *S*. *mobaraensis*. Highest amounts of Sml-1 were already obtained after 48 hours of culture when TGase activity was still increasing (Fig 5a). Secretion of Sml-1 occurred like the other substrates of TGase in an early growth phase. Degradation of the enzyme in the further course of culture indicated the observed sensitivity of Sml-1 against intrinsic proteases. Surface colonies were used to determine the occurrence of Sml-1 during unimpaired growth of *S*. *mobaraensis* for several days. Overgrown agar plates were covered with nitrocefin every day and incubated for 30 minutes to form the red hydrolysis product. Colony staining on the first day showed again the early export of the Sml-1 β-lactamase. After two additional days, more than half of the plate culture was colored red (Fig 5b). Maximum in β-lactamase activity was found on day five as increase in absorbance of plate extracts indicated (S8 Fig).

### Properties and kinetic parameters of the beta-lactamase Sml-1

The recombinant *r*Sml-1 was further characterized to determine influence of pH, temperature and metal ions on enzyme activity and protein stability. The highest activity was monitored at pH 7 and 45°C which complies with data from other enzymes of the mesophilic bacterium such as transglutaminase (S5 Fig). Buffer substances such as citrate, phosphate, Tris-HCl and glycine did not modify considerably protein conformation. Effect of metals on *r*Sml-1 was likewise low, and activity was not more enhanced than ten percent by 50 mM Na^+^, 10 μM Ca^2+^, and 50-200 nM Zn^2+^. It is evident that Sml-1 is not a Zn^2+^-dependent β-lactamase (Table 1).

**Table 1:** Kinetic parameters of various β-lactamases using nitrocefin as substrate.

β-Lactamase	Organism	Assay conditions	K_m_	k_cat_	k_cat_/K_m_
			(μM)	(s^-1^)	(mM^-1^ s^-1^)
*r*Sml-1	*S*. *mobaraensis*	50 mM Tris pH 7;50 mM NaCl; 28°C	94.3±1.8 ^a^^)^	0.39±0.03 ^a^^)^	4.1
Class A ^b^^)^	*S*. *cacaoi*	50 mM phosphate pH 7;30°C	475±50	307±32 ^c^^)^	646 ^c^^)^
Class B ^d^^)^	*A*. *hydrophila*	50 mM cacodylate pH 7;0.1 mM ZnCl_2_; 30°C	100±8	0.31	3.1
Class B ^d^^)^	*B*. *cereus*	50 mM cacodylate pH 7; 0.1 mM ZnCl_2_; 30°C	70±5	45	642
Class B ^d^^)^	*P*. *maltophilia*	50 mM cacodylate pH 7; 0.1 mM ZnCl_2_; 35°C	7±1	20	2857
Class C ^e^^)^	*C*. *freundii*	10 mM Hepes pH 8.2; 0.2 M NaCl; 50 μg/ml BSA	12±1	330±20	27500

^a^ Mean of three independent measurements.

^b^ Ref. [18].

^c^ Calculated from published data [18].

^d^ Ref. [19].

^e^ Ref. [17].

The kinetic parameters were determined at pH 7 using 60–200 μM nitrocefin and 3.5 μM *r*Sml-1 at 28°C (S4 Fig). Affinity of the enzyme for nitrocefin (K_m_ of 94.3 μM) was comparable with that of the Zn^2+^-dependent β-lactamases from *Aeromonas hydrophila* or *Bacillus cereus* [19] (Table 1). The Michaelis constant of the *S*. *cacaoi* enzyme, a class A β-lactamase, was considerably higher, even reflecting the low sequence identity of 18% between both *Streptomyces* proteins [18]. In contrast, *Pseudomonas maltophilia* and *Citrobacter freundii* have evolved class B and class C β-lactamases exhibiting active sites more suited to attract nitrocefin. Compared with *r*Sml-1, published K_m_ values differ by one order of magnitude [17,19]. Moreover, the β-lactamases from *S*. *mobaraensis* (k_cat_ of 0.39 s^-1^ for *r*Sml-1) and *A*. *hydrophila* hydrolyze nitrocefin slowly as indicated by turnover rates that are three orders of magnitude lower than those of the most effective enzymes.

Zinc-independent β-lactamases exhibit an essential serine residue in the active site which is part of a SXXK motif and even present in the sequence of Sml-1 (S96ITK, *cf*. Fig 2). The question arose whether *r*Sml-1 should be inhibited by serine and cysteine protease inhibitors. Incubation was performed using PMSF, AEBSF, TLCK, leupeptin, N-ethyl maleinimide, and iodoacetamide, respectively, in concentrations of 1 mM each. Besides, *r*Sml-1 was combined with 1 mM EDTA and up to 1.5 mM Ac_2_Lys-DAla-DAla to confirm independence from Zn^2+^ and lack of DD-carboxypeptidase activity. In all cases, the effect of the inhibitory molecules was negligible. In contrast, the β-lactam antibiotics penicillin G and ampicillin reduced Sml-1 activity considerably. The obtained half maximal inhibitory concentrations (I_50_) for penicillin G and ampicillin were 0.27±0.01 mM (K_i_ of 0.1 mM) and 0.48±0.03 mM (K_i_ of 0.18 mM), respectively (S9 Fig).

### Putative active site and classification of the beta-lactamase Sml-1

Serine β-lactamases and LMM-PBP involved in cell wall turnover have similar structures and share conserved sequence motifs which form the active site (Table 2). SXXK (motif 1), (S,Y)X(N,C) (motif 2), and (K,H)(T,S)G (motif 3) are described to be essential for nucleophilic attack, proton transfer, and substrate binding [20]. The glutamate of EXXLN, an additional motif referred to as motif 4 in Table 2, is oriented to the active site in class A β-lactamases [21]. Both variants of motif 2 and motif 3, *i*. *e*. SXX/S(Y)XN and KTG/HXG, respectively, are present in the sequence of Sml-1 (Table 2 and Fig 2), even though only one is needed for catalysis and substrate binding.

**Table 2:** Sequence motifs forming the active site of β-lactamases and penicillin-binding proteins as described by Ghuysen [20] with some modifications.

Enzyme	Motif 1SXXK	Motif 2SXX	Motif 2’S(Y)XN	Motif 4EXXLN	Motif 3KTG	Motif 3’HXG
*Streptomyces mobaraensis*Sml-1	S96ITK	S149GV	Y201SN	absent	K317TG	H342DG
*Streptomyces* sp. K15Class A LMM-PBP	S64TTK	S125GC	absent	absent	K242TG	absent
*Streptomyces sp*. R61Class C LMM-PBP	S93VTK	S141GL	Y190SN	absent	absent	H329TG
*Actinomadura* sp. R39Class C LMM-PBP	S98NMK	S162GV	S347NN	E377EALS	K459TG	H489SG
*Streptomyces albus* GClass A β-lactamase	S89VFK	absent	S155DN	E191PELN	K259TG	absent
*Streptomyces cacaoi*Class A β-lactamase	S93TFK	S138PV	S162DN	E198QELG	K270SG	H300GD
*Citrobacter freundii*Class C β-lactamase	S84VSK	absent	Y170AN	absent	K335TG	absent
*Salmonella typhimurium*Class D β-lactamase	S72TFK	S120TV	Y146GN	E161GSLA	K210TG	absent

Numbering includes the amino acids of the signal peptides. Residues pointing into the active site are underlined.

**Fig 6:**
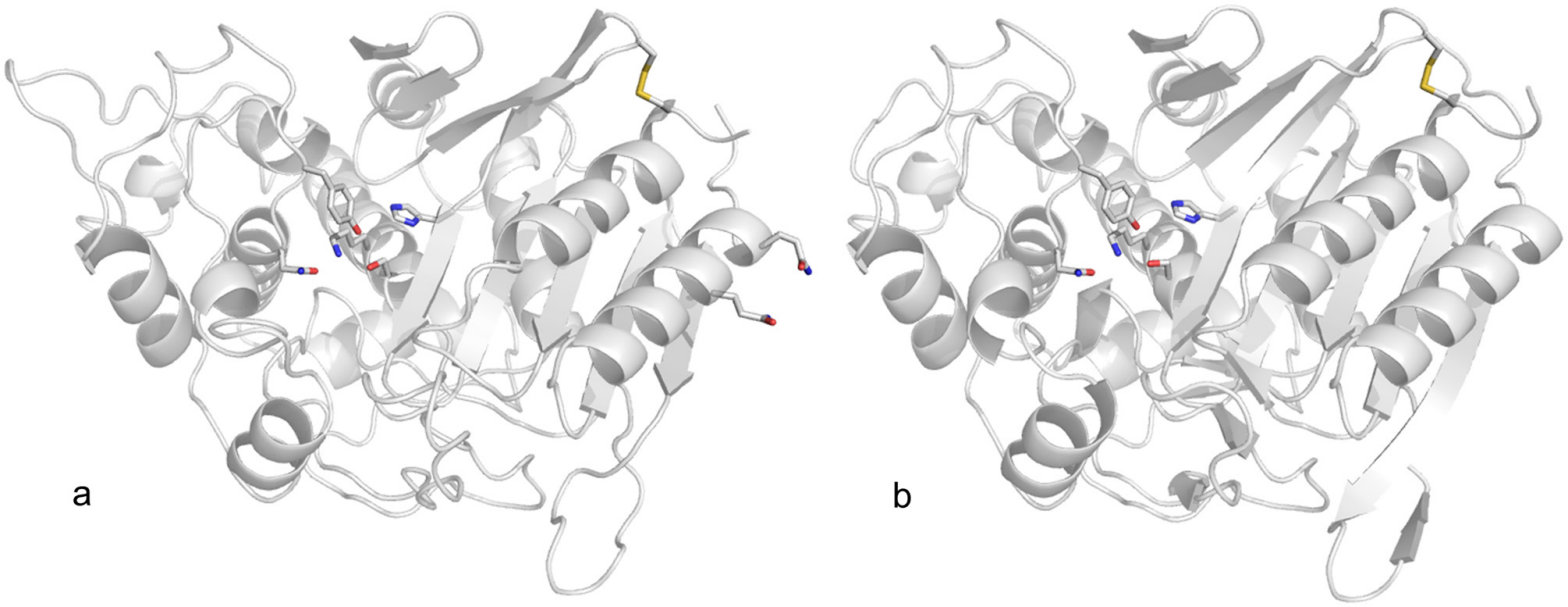
Hypothetical structure (a) of the β-lactamase Sml-1 from *S*. *mobaraensis*. For comparison, tertiary structure of the DD-transpeptidase from *Streptomyces* sp. R61 is shown in addition (b). Putative active site residues (R61 residues in brackets) are Ser96 (Ser93), Lys99 (Lys96), Tyr201 (Tyr190), Asn201 (Asn192) and His342 (His329). Structures even indicate cysteines at the C-termini forming a single disulphide bridge in R61 and two exposed glutamines of two N-terminal Q pairs of Sml-1. Structure of Sml-1 was modelled and visualized using SwissProt and PyMol.

The serine β-hydroxyl function of motif 1 is always described as the nucleophile attacking the β-lactam carboxyl carbon atom while motif 1 lysine, motif 2 serine or tyrosine, and motif 4 glutamate are thought to act as general bases. The lysine or histidine of motif 3 only seems to contribute to substrate binding. In general, sequence attributes are not sufficient to assess type and activity of a given β-lactamase or PBP [22]. This was the reason why a homology model was generated *via* secondary structure alignment to classify the enzyme Sml-1 and its tertiary structure (Fig 6a).

The hypothetical structure of Sml-1 revealed five active site residues in an helical environment properly separated from both terminal helices by an eight-stranded β-sheet. Ser96 and Lys99 of motif 1 are located in helix α2, Tyr201 and Asn203 in an extended loop within the global helical domain, and His342 in strand β6 (Fig 6a and Table 2). The arrangement strongly resembles the tertiary structure of the DD-transpeptidase from *Streptomyces* sp. R61 (Fig 6b) which is in turn close to class C β-lactamase foldings [23]. The putative structure suggests that Sml-1 belongs more to class C than to class A or class D β-lactamases.

## Discussion

The present work was performed to uncover novel substrates of microbial transglutaminase (TGase) from *S*. *mobaraensis* and to illuminate their roles in bacterial life. During submerged culture growth, substrates of TGase such as the dispase-autolysis inducing protein DAIP, the *Streptomyces* subtilisin and TAMP inhibitor SSTI or the *Streptomyces* papain inhibitor SPI are exported earlier than TGase, and protein cross-linking is mostly prevented by shearing forces (Fig 1). If *S*. *mobaraensis* secretes additional substrates, they should emerge under the same culture conditions that are used for the production of TGase. The extracellular proteins can be readily precipitated by ethanol without loss in activity, and the procedure allows complete separation of the substrates from the cross-linking enzyme [8,9]. Screening of the extracellular proteins from *S*. *mobaraensis* yielded an occurrence of the novel β-lactamase Sml-1 (M3B5M4) in an early growth phase losing solubility by the addition of at least 50 vol% ethanol. The amino acid sequence assigned Sml-1 to the large family of low molecular mass penicillin-binding proteins (LMM-PBP) that comprise besides penicillin-binding proteins β-lactamases, DD-carboxypeptidases, DD-transpeptidases, and peptidoglycan peptidases. Missing proteolytic activity and failure to truncate D-alanine from the amino-protected tripeptide Ac_2_Lys-DAla-DAla ruled out that Sml-1 participates in cell wall turnover. However, the enzyme hydrolyzed the cephalosporin analogue nitrocefin, thus emphasizing the more protective character in inactivating β-lactam antibiotics. Highest activity was determined at pH 7 and moderate temperatures reflecting the growth style of a mesophilic bacterium in a neutral environment. Metals such as zinc, calcium and sodium hardly influenced enzyme activity, even showing that Sml-1 is not a class B β-lactamase. Compared with other β-lactamases, in particular distinct class B and class C β-lactamases, affinity of *r*Sml-1 for nitrocefin was low. The same applies for the turnover rate differing from others by up to three orders of magnitude. Moreover, weak inhibition of nitrocefin hydrolysis by penicillin G and ampicillin confirmed that Sml-1 is a moderate penicillin-interactive protein cleaving slowly the amide bond of the four-membered β-lactam ring.

The β-lactamase Sml-1 from *S*. *mobaraensis* shares amino acid sequence identities between 49 percent and 54 percent to the most related *Streptomyces* proteins (e. g. D9WQ26, D9X6B6, S4N406), among them a putative peptidase from *S*. *mobaraensis* (M3C7Q3). The considerably lower similarity to the well characterized β-lactamases from *S*. *cacaoi* and *S*. *albus* G [24] can be explained by the fact that Sml-1 is rather a class C than a class A β-lactamase. Curiously, structures of the modelled Sml-1 and the DD-carboxypeptidase from *Streptomyces* sp. R61 are quite similar, although Sml-1 could not cleave the R61 substrate Ac_2_Lys-DAla-DAla. In the past, several authors have referred to the high flexibility of PBP structures. Substrate and protonation state of the residues involved in catalysis may induce different conformations. Variability was studied in a more recent work indicating at least four conformations for the ester carboxyl group of the acyl-enzyme intermediate between aztreonam and the class C β-lactamase from *C*. *freundii* by infrared difference spectroscopy [25]. Such conformational variety hardly allows general conclusions about the active site without crystal structures, even in complex with β-lactam antibiotics.

That the heterologously produced β-lactamase *r*Sml-1 is a substrate of TGase was clearly proven by the incorporation of biotinylated lysine and glutamine equivalents, even showing cross-linked aggregates. Two N-terminal glutamine pairs in the Sml-1 sequence are particularly striking (Fig 2). One of each protrudes from helix 1 in the putative Sml-1 structure (Fig 6). A peptide containing both Q pairs was synthesized and proved to be an excellent substrate of TGase (Kolmar, personal communication).

The novel β-lactamase Sml-1 from *S*. *mobaraensis* fits well to the already characterized transglutaminase substrates such as SSTI, SPI and DAIP suggesting a similar role in bacterial life. Aerial hyphae of streptomycetes are developing saprophytically onto the mature substrate mycelium in an apoptosis-like process [26]. Growth into the air is supported by the amphiphilic lanthionine SapB [27], and the growing peptidoglycan cell wall is covered by an amphiphilic protein sheath consisting of chaplins and rodlins [28,29]. We assume that the substrates of TGase, characterized so far, are evolved by *S*. *mobaraensis* and other TGase producing streptomycetes to ensure undisturbed growth and to protect more efficiently the outer protein layer against proteolytic degradation. While DAIP, SPI and SSTI inhibit a broad spectrum of microbial endoproteases, in particular from bacilli, the β-lactamase is enabled to trap and inactivate penicillin- and cephalosporin-like compounds. Such antibiotics may be produced by nutrient competitors. However, distinct streptomycetes such as *S*. *clavuligerus* even secrete β-lactams [30]. It may be equally conceivable at present that *S*. *mobaraensis* needs Sml-1 to encounter the own biological weapons.

## Supporting Information

S1 Fig Purification of the 39 kDa protein (β-lactamase Sml-1) by Fractogel EMD SO_3_¯ chromatography. Insert: protein pattern of the top fractions of peak 2 and peak 3.
(TIF)

S2 Fig Optimized gene sequence encoding the β-lactamase Sml-1 from *S*. *mobaraensis* for the production in *E*. *coli*. Modified codons are shown in red.
(TIF)

S3 Fig Purification of the 39 kDa protein (β-lactamase Sml-1) from *S*. *mobaraensis* produced in *E*. *coli* BL21(DE3) RIL by IMAC. Insert: protein pattern of the top fractions of flowthrough (1) and the peaks 2/3.
(TIF)

S4 Fig Hydrolysis of nitrocefin by the β-lactamase Sml-1 from *S*. *mobaraensis*. The reaction mediated by 3.5 μM Sml-1 in 50 mM Tris/HCl containing 50 mM NaCl were monitored at pH 7 for the indicated times and wave-length. The nitrocefin concentrations were 0 μM (pigeon blue), 60 μM (orange), 80 μM (light-blue), 100 μM (purple), 120 μM (green), 160 μM (Bordeaux red), and 200 μM (dark-blue).
(TIF)

S5 Fig Influence of pH (a) and temperature (b) on β-lactamase activity. The pH optimum was determined by monitoring continuously *r*Sml-1 (1.6 μM) mediated hydrolysis of 0.1 mM nitrocefin in 50 mM buffer containing 50 mM NaCl up to 10 min at 492 nm (5.2 mU/ml = 100%). The used buffers were citrate (3–6.5, dark-blue), phosphate (6–8, green), Tris/HCl (7–9, purple), and glycine (9–10, light-blue). The temperature optimum (blue) was measured using Tris/HCl pH 7 (9.9 mU/ml = 100%). Controls without *r*Sml-1 are shown in red. All data are means of two independent measurements.
(TIF)

S6 Fig Transglutaminase mediated biotinylation of the recombinant 39 kDa protein (β-lactamase Sml-1) from *S*. *mobaraensis*. (a) Biotin blots showing streptavidin alkaline phosphatase stained *r*p39 linked to 1-*N*-biotinyl-6-*N’*-(carbobenzoxy-L-glutaminylglycyl)diamidohexane (labelling of lysines) and monobiotinylcadaverine (labelling of glutamines). Lanes 1/1’, labelling mixtures; lanes 2/2’, 3/3’, and 4/4’, controls without TGase, *r*p39 or the biotinylated probes, respectively. (b) Structures of biotinylated Sml-1.
(TIF)

S7 Fig Separation of the 39 kDa protein (β-lactamase Sml-1) from SPI and SSTI by Fractogel EMD TMAE chromatography. Insert: protein pattern of the top fractions 1-3.
(TIF)

S8 Fig Activity of the β-lactamase Sml-1 in surfaces colonies of *S*. *mobaraensis*. Overgrown agar plates were covered for 15 min (blue) and 30 min (red) using 0.15 mM nitrocefin. Increase in absorbance of nitrocefin supernatant aliquots was determined at 492 nm.
(TIF)

S9 Fig Inhibition of the β-lactamase Sml-1 by penicillin G and ampicillin. The reaction mixtures contained besides ampicillin (blue) and penicillin G (red) 0.2 mM nitrocefin, 4 μM Sml-1, 50 mM NaCl and 50 mM Tris/HCl pH 7.
(TIF)

## Abbreviations

DAIP: dispase autolysis inducing protein
PBP: penicillin-binding protein
Sml-1: beta-lactamase-1 from *Streptomyces mobaraensis*
SPI: *Streptomyces* papain inhibitor
SSTI: *Streptomyces* subtilisin and TAMP inhibitor
TAMP: transglutaminase activating metalloprotease
TGase: transglutaminase
