# Supplementary figures and images for: Involvement of a Novel Class C Beta-Lactamase in the Transglutaminase Mediated Cross-Linking Cascade of Streptomyces mobaraensis DSM 40847
Source: PLoS One. 2016 Feb 17;11(2):e0149145. doi: 10.1371/journal.pone.0149145 (PMC4757070; doi:10.1371/journal.pone.0149145)

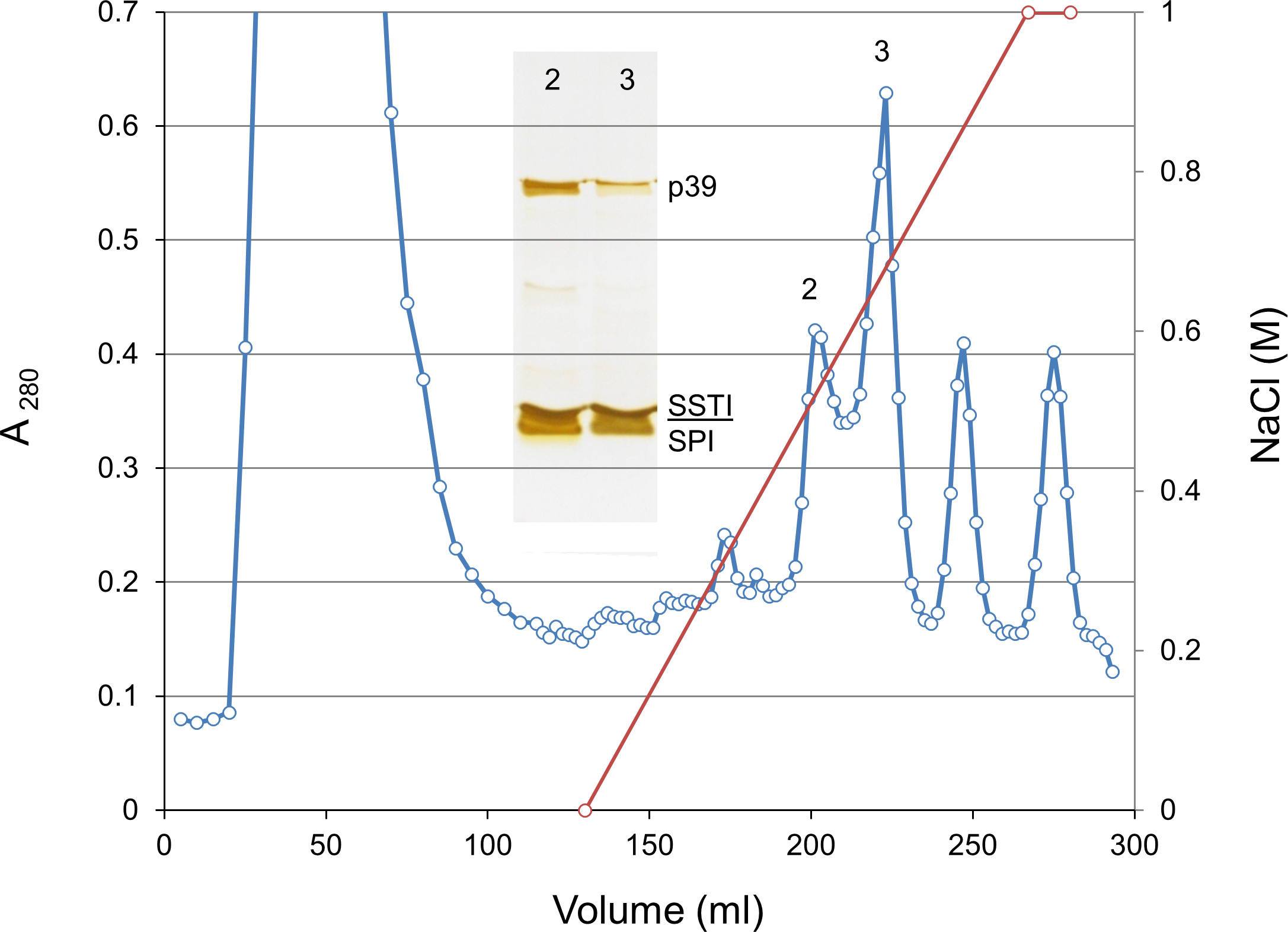

Supplement: S1 Fig — Insert: protein pattern of the top fractions of peak 2 and peak 3. (TIF) [file pone.0149145.s001.tif]

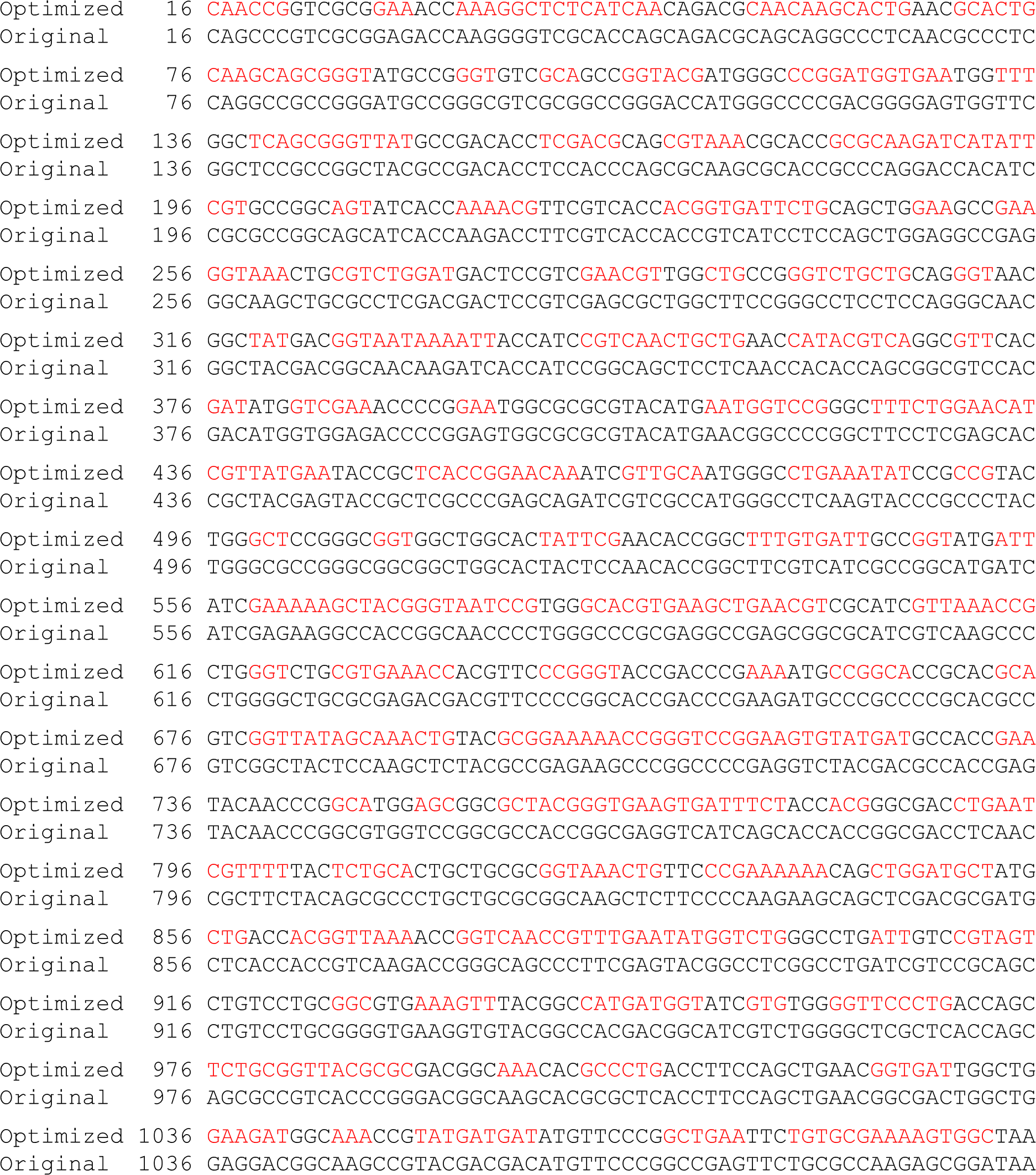

Supplement: S2 Fig — Modified codons are shown in red. (TIF) [file pone.0149145.s002.tif]

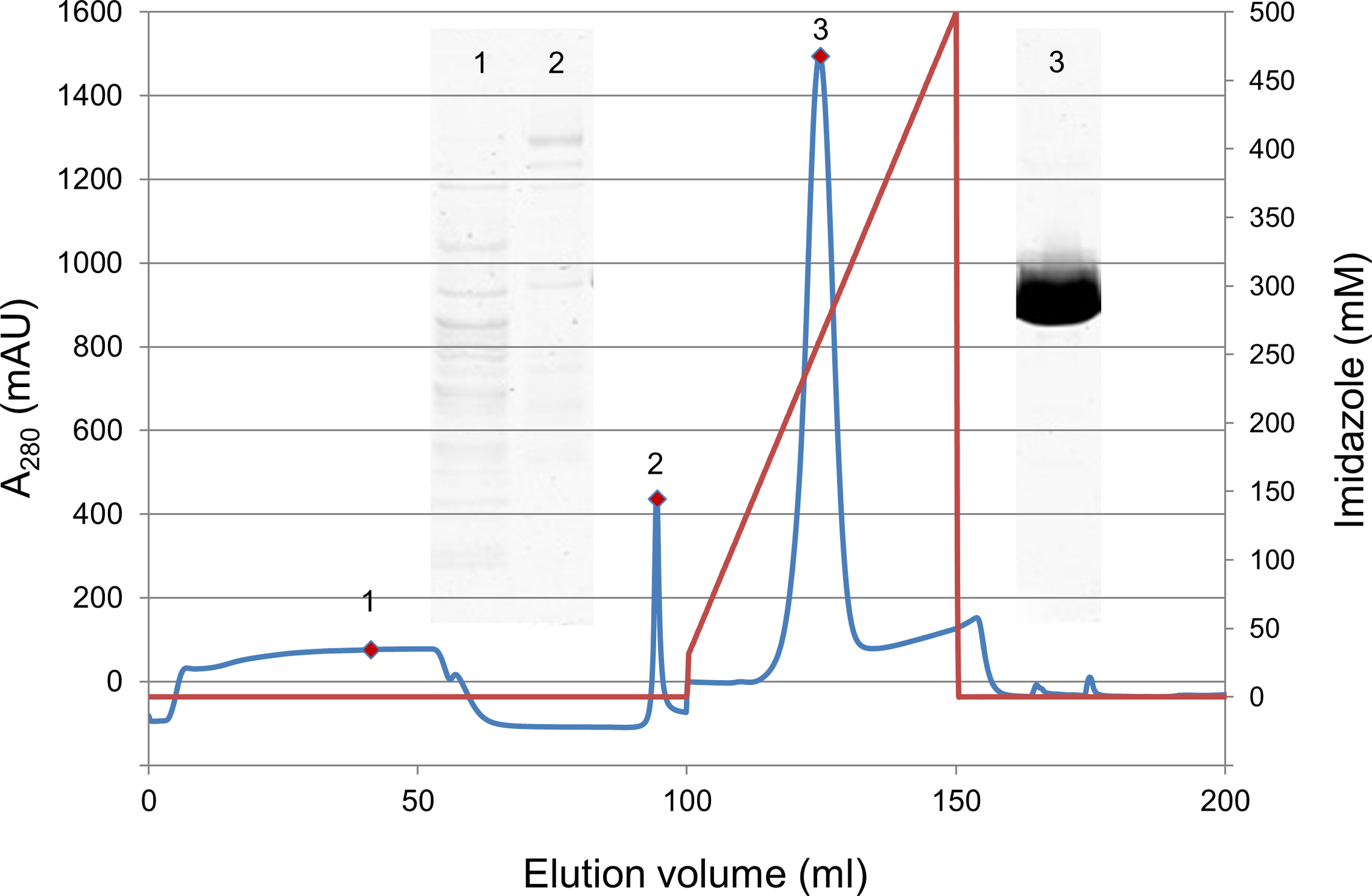

Supplement: S3 Fig — Insert: protein pattern of the top fractions of flowthrough (1) and the peaks 2/3. (TIF) [file pone.0149145.s003.tif]

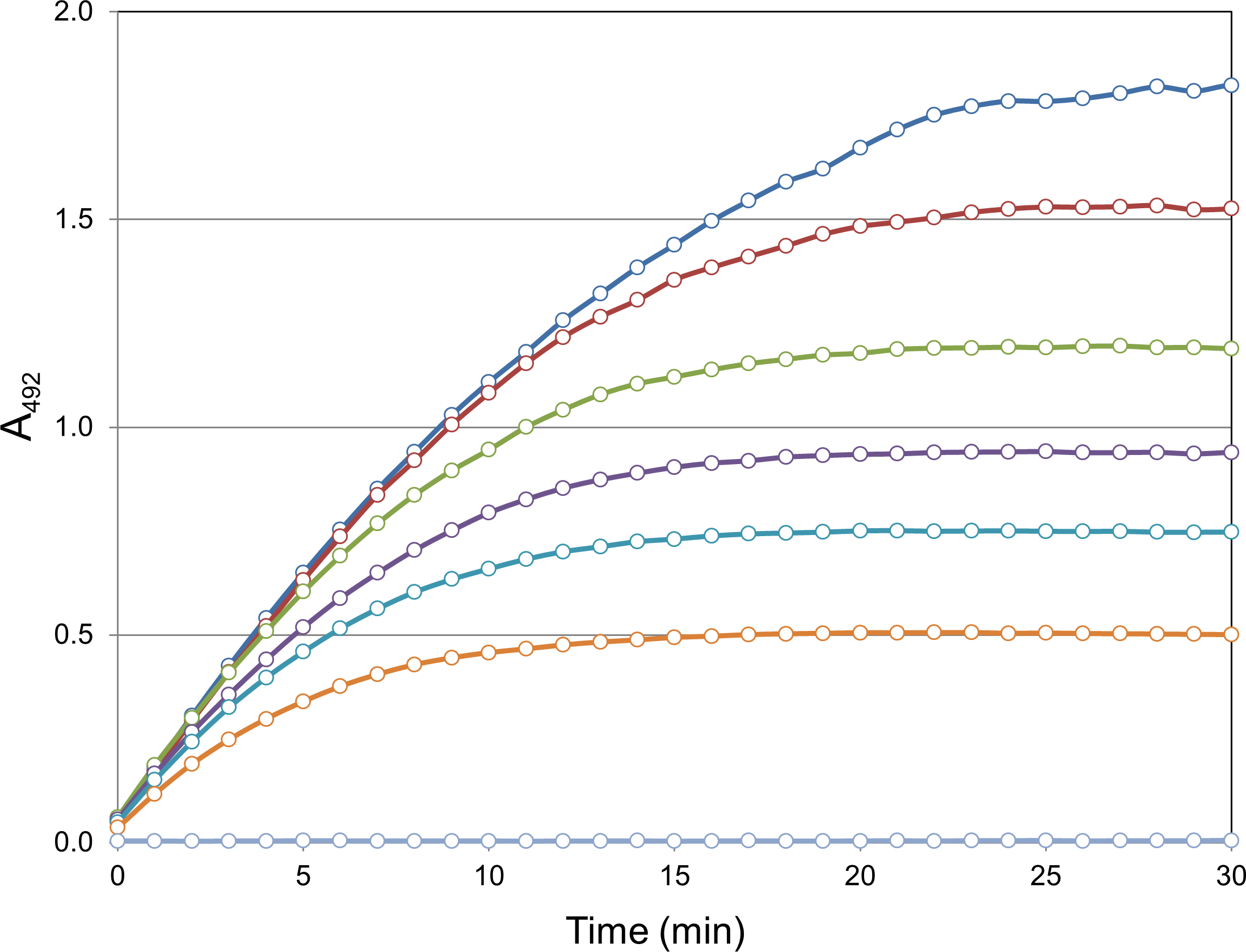

Supplement: S4 Fig — The reaction mediated by 3.5 μM Sml-1 in 50 mM Tris/HCl containing 50 mM NaCl were monitored at pH 7 for the indicated times and wave-length. The nitrocefin concentrations were 0 μM (pigeon blue), 60 μM (orange), 80 μM (light-blue), 100 μM (purple), 120 μM (green), 160 μM (Bordeaux red), and 200 μM (dark-blue). (TIF) [file pone.0149145.s004.tif]

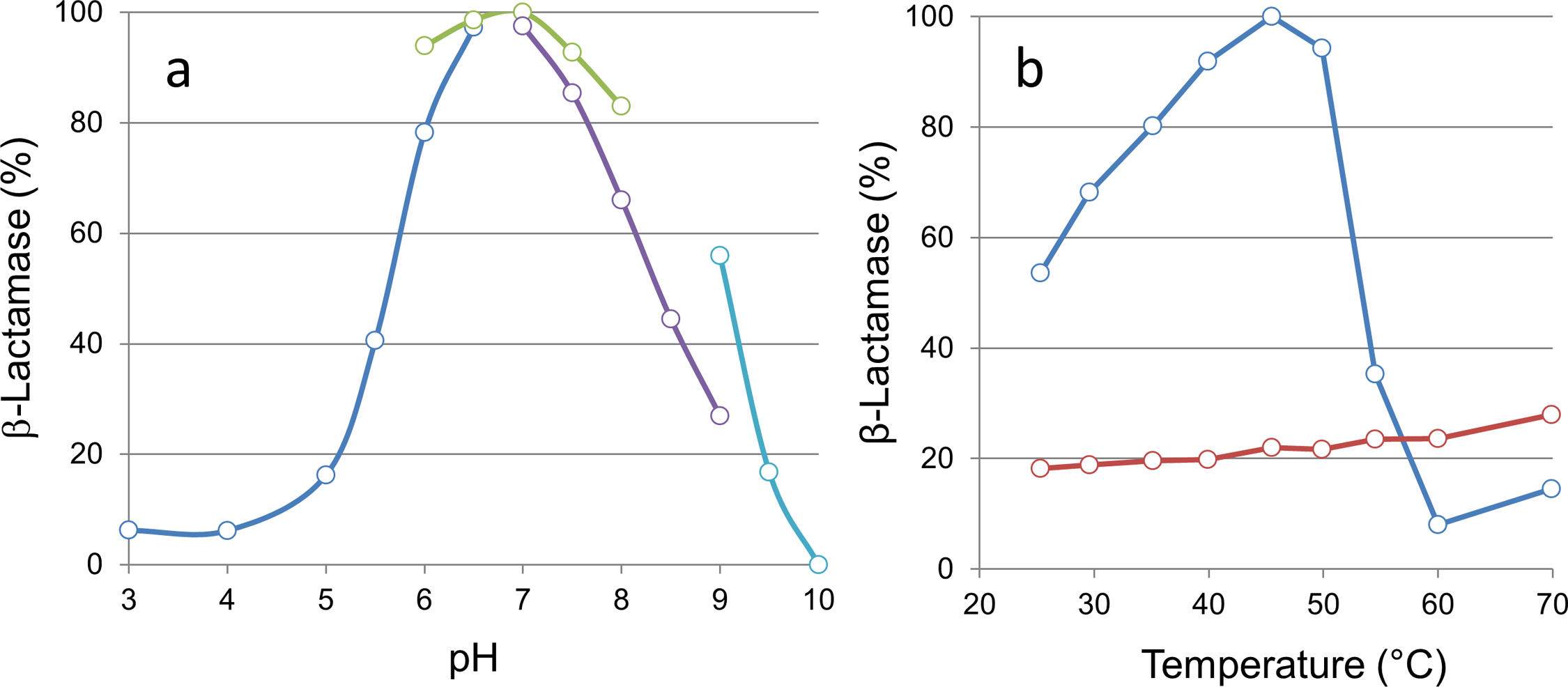

Supplement: S5 Fig — The pH optimum was determined by monitoring continuously rSml-1 (1.6 μM) mediated hydrolysis of 0.1 mM nitrocefin in 50 mM buffer containing 50 mM NaCl up to 10 min at 492 nm (5.2 mU/ml = 100%). The used buffers were citrate (3–6.5, dark-blue), phosphate (6–8, green), Tris/HCl (7–9, purple), and glycine (9–10, light-blue). The temperature optimum (blue) was measured using Tris/HCl pH 7 (9.9 mU/ml = 100%). Controls without rSml-1 are shown in red. All data are means of two independent measurements. (TIF) [file pone.0149145.s005.tif]

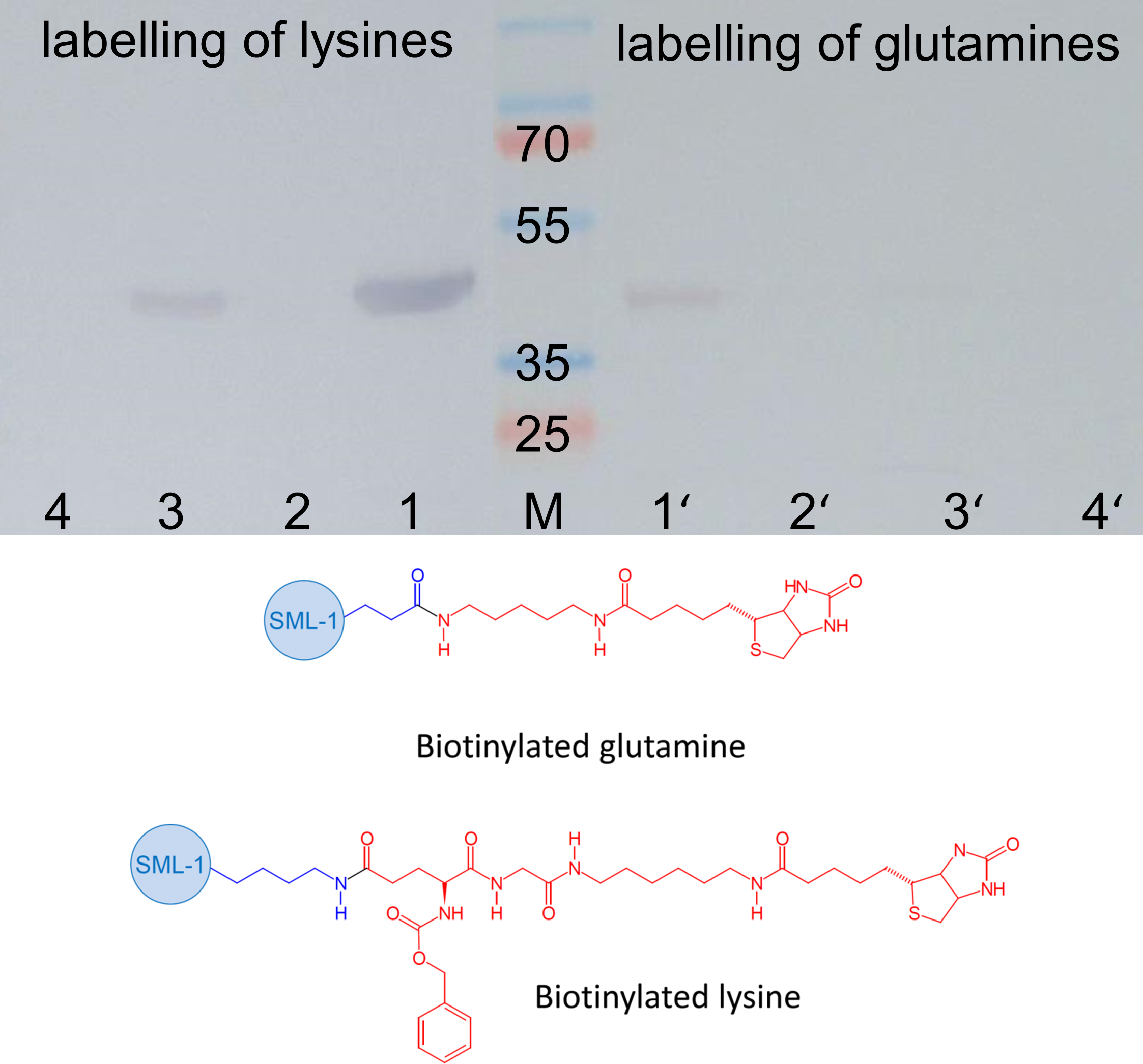

Supplement: S6 Fig — (a) Biotin blots showing streptavidin alkaline phosphatase stained rp39 linked to 1-N-biotinyl-6-N’-(carbobenzoxy-L-glutaminylglycyl)diamidohexane (labelling of lysines) and monobiotinylcadaverine (labelling of glutamines). Lanes 1/1’, labelling mixtures; lanes 2/2’, 3/3’, and 4/4’, controls without TGase, rp39 or the biotinylated probes, respectively. (b) Structures of biotinylated Sml-1. (TIF) [file pone.0149145.s006.tif]

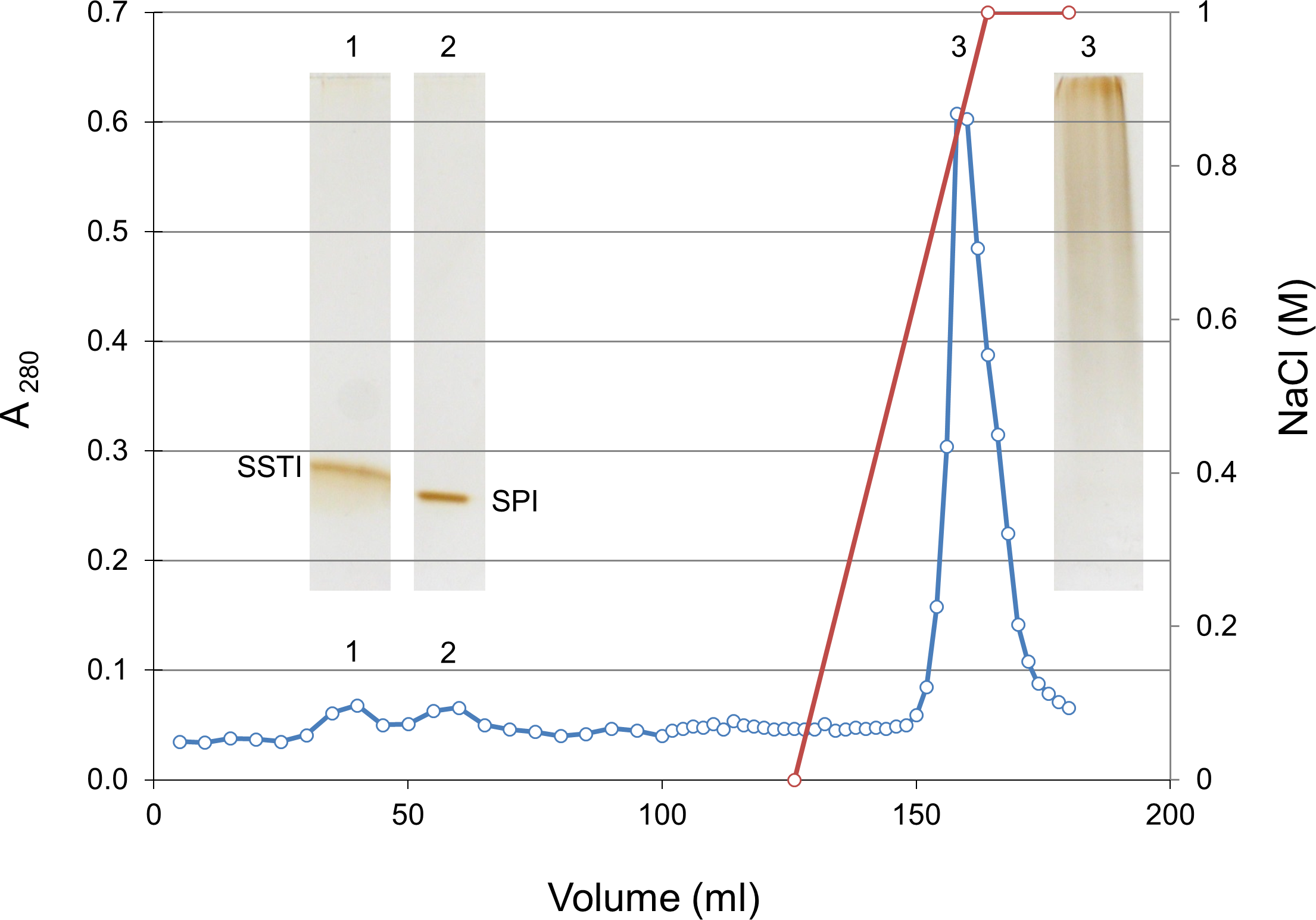

Supplement: S7 Fig — Insert: protein pattern of the top fractions 1-3. (TIF) [file pone.0149145.s007.tif]

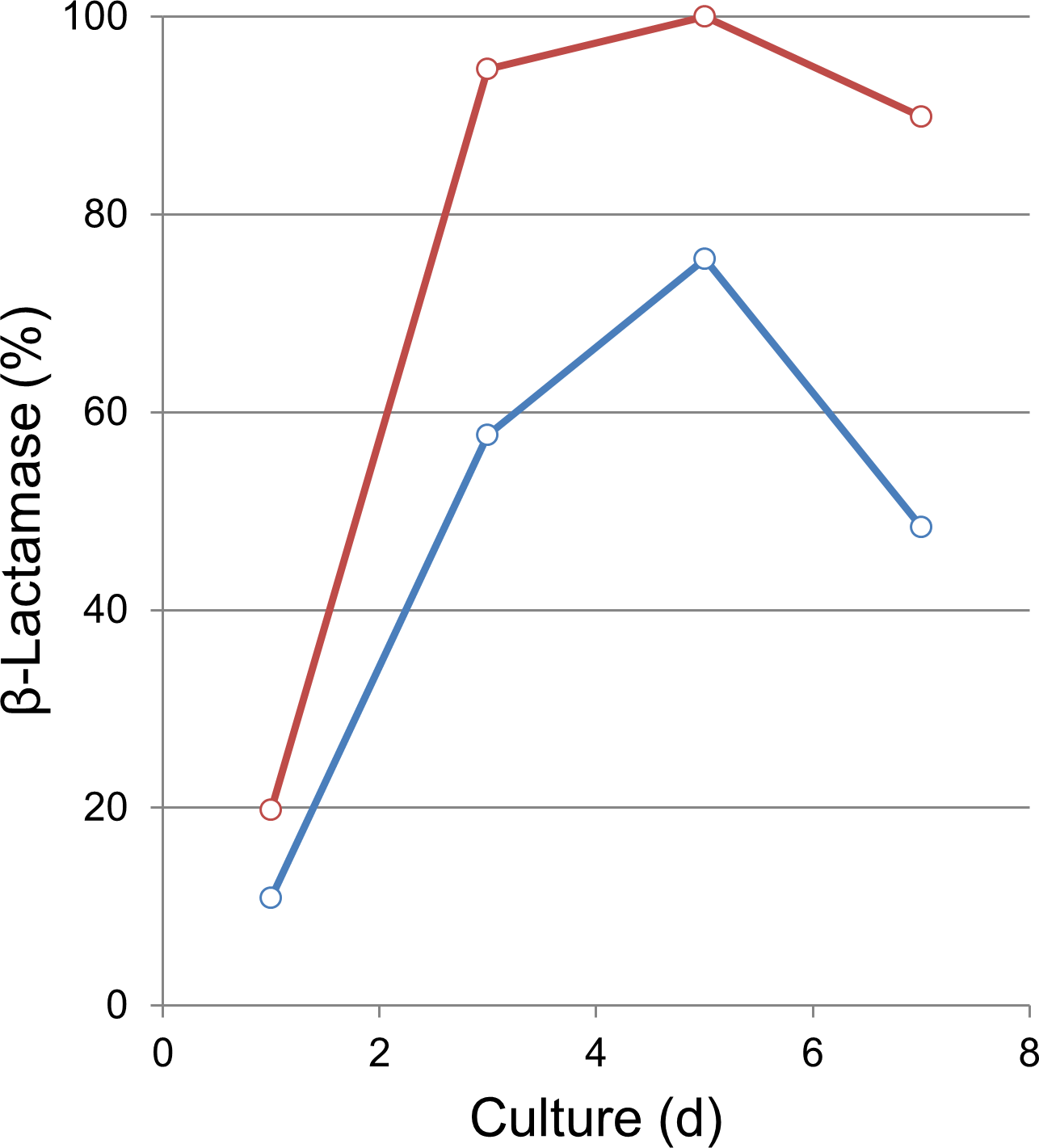

Supplement: S8 Fig — Overgrown agar plates were covered for 15 min (blue) and 30 min (red) using 0.15 mM nitrocefin. Increase in absorbance of nitrocefin supernatant aliquots was determined at 492 nm. (TIF) [file pone.0149145.s008.tif]

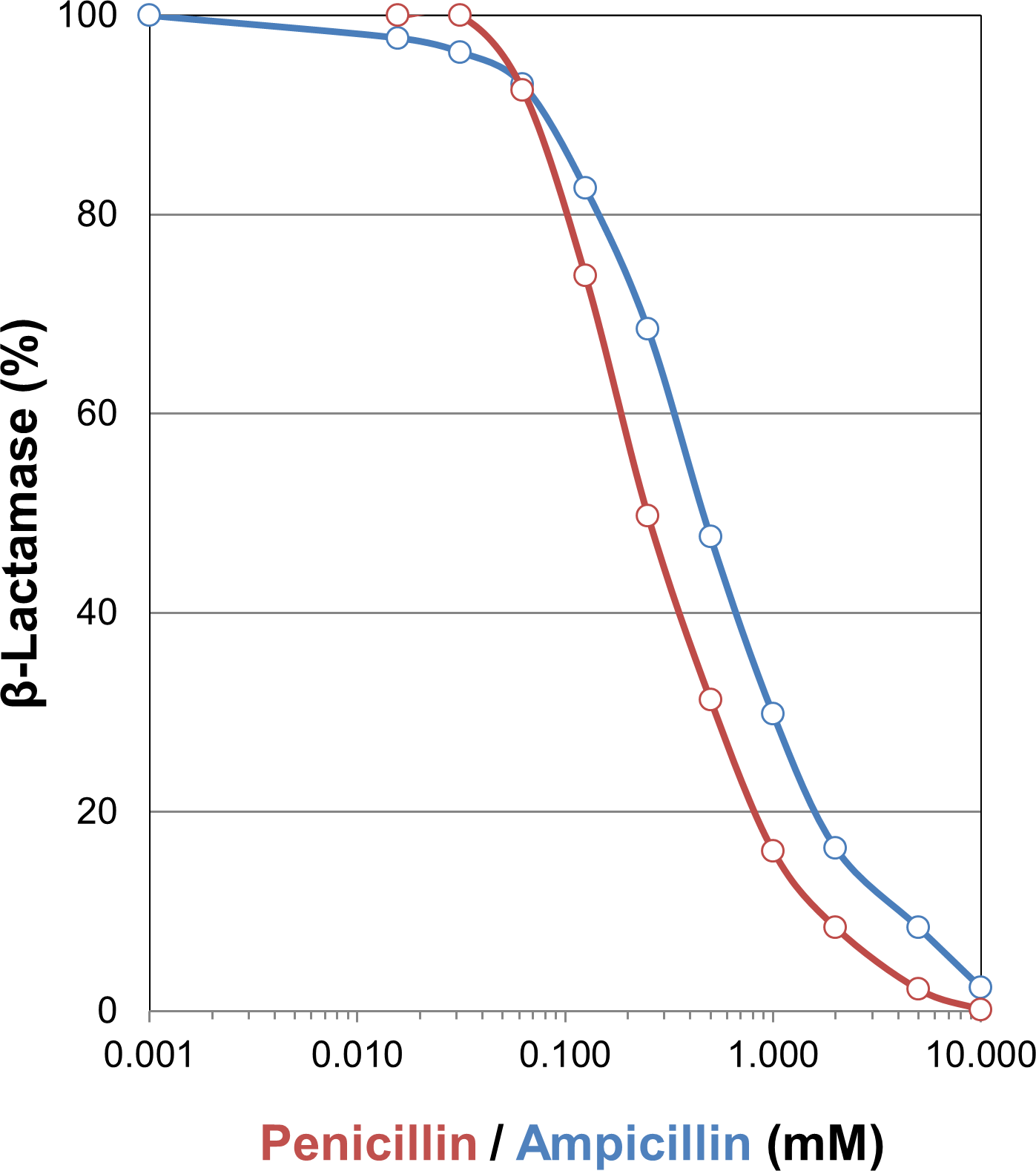

Supplement: S9 Fig — The reaction mixtures contained besides ampicillin (blue) and penicillin G (red) 0.2 mM nitrocefin, 4 μM Sml-1, 50 mM NaCl and 50 mM Tris/HCl pH 7. (TIF) [file pone.0149145.s009.tif]
